# Designing a childhood obesity preventive intervention using the multiphase optimization strategy: The Healthy Bodies Project

**DOI:** 10.1177/17407745231167115

**Published:** 2023-04-19

**Authors:** Lori A Francis, Robert L Nix, Rhonda BeLue, Kathleen L Keller, Kari C Kugler, Brandi Y Rollins, Jennifer S Savage

**Affiliations:** 1 Department of Biobehavioral Health, College of Health and Human Development, The Pennsylvania State University, University Park, PA, USA; 2 Department of Human Development and Family Studies, School of Human Ecology, University of Wisconsin-Madison, Madison, WI, USA; 3 Department of Public Health, College for Health, Community and Policy, University of Texas at San Antonio, San Antonio, TX, USA; 4 Department of Nutritional Sciences, College of Health and Human Development, The Pennsylvania State University, University Park, PA, USA

**Keywords:** Multiphase optimization strategy, obesity prevention, healthy eating, physical activity, self-regulation, parenting

## Abstract

**Background/Aims:**

Preventing the development of childhood obesity requires multilevel, multicomponent, comprehensive approaches. Study designs often do not allow for systematic evaluation of the efficacy of individual intervention components before the intervention is fully tested. As such, childhood obesity prevention programs may contain a mix of effective and ineffective components. This article describes the design and rationale of a childhood obesity preventive intervention developed using the multiphase optimization strategy, an engineering-inspired framework for optimizing behavioral interventions. Using a series of randomized experiments, the objective of the study was to systematically test, select, and refine candidate components to build an optimized childhood obesity preventive intervention to be evaluated in a subsequent randomized controlled trial.

**Methods:**

A 2^4^ full factorial design was used to test the individual and combined effects of four candidate intervention components intended to reduce the risk for childhood obesity. These components were designed with a focus on (a) improving children’s healthy eating behaviors and nutrition knowledge, (b) increasing physical activity and reducing sedentary activity in the childcare setting, (c) improving children’s behavioral self-regulation, and (d) providing parental web-based education to address child target outcomes. The components were tested with approximately 1400 preschool children, ages 3–5 years in center-based childcare programs in Pennsylvania, the majority of which served predominantly Head-Start eligible households. Primary child outcomes included healthy eating knowledge, physical and sedentary activity, and behavioral self-regulation. Secondary outcomes included children’s body mass index and appetitive traits related to appetite regulation.

**Results:**

Four intervention components were developed, including three classroom curricula designed to increase preschool children’s nutrition knowledge, physical activity, and behavioral, emotional, and eating regulation. A web-based parent education component included 18 lessons designed to improve parenting practices and home environments that would bolster the effects of the classroom curricula. A plan for analyzing the specific contribution of each component to a larger intervention was developed and is described. The efficacy of the four components can be evaluated to determine the extent to which they, individually and in combination, produce detectable changes in childhood obesity risk factors. The resulting optimized intervention should later be evaluated in a randomized controlled trial, which may provide new information on promising targets for obesity prevention in young children.

**Conclusion:**

This research project highlights the ways in which an innovative approach to the design and initial evaluation of preventive interventions may increase the likelihood of long-term success. The lessons from this research project have implications for childhood obesity research as well as other preventive interventions that include multiple components, each targeting unique contributors to a multifaceted problem.

## Introduction

Approximately, 12% of US children, ages 2–5, are classified with obesity, with the prevalence increasing with age,^1^ and with children from low-income households being disproportionately impacted.^2,3^ Due to the challenges of reversing childhood obesity once it has developed, comprehensive prevention approaches that reduce children’s obesogenic behaviors in early childhood, and confer future protection, hold promise for influencing lifelong habits.

### Optimizing childhood obesity prevention programs using an engineering-inspired framework

Combating childhood obesity requires multilevel, multicomponent approaches. A review^4^ of early childhood obesity prevention programs showed that < 50% of the childcare-based programs reviewed produced significant effects on children’s body mass index (BMI). Efficacious programs intervened on preschool children’s physical activity,^5–8^ or on multiple levels of influence, including children’s nutrition and physical activity, parent behaviors, the childcare provider, and/or childcare policies.^9,10^ Efficacious, multicomponent programs with preschoolers from lower income households produced significant effects on food parenting practices,^11^ and on parents’ reports of children’s self-regulation and sugar-sweetened beverage intake,^12^ but they had no effect on child weight status. Childhood obesity prevention study designs often do not allow for systematic testing of the efficacy of individual intervention components before the intervention is fully tested. As such, these programs may contain a mix of effective and ineffective components, which may partly explain limited success.

Typically, childhood obesity prevention programs are tested in a classic randomized controlled trial (RCT), such that, all of the possible components are tested at once, and the intervention is deemed effective or efficacious if the intervention group exhibited better outcomes than the control or comparison group. If the intervention is not deemed efficacious, a new RCT is developed, with new or different combinations of components, and the process is repeated. What we do not know at the end of the RCT, however, is *which* components were the most efficacious, in *what combinations*, and *which* components were unnecessary. Innovative approaches, such as the multiphase optimization strategy (MOST),^13^ can inform the design of more resource-efficient, efficacious, and scalable obesity prevention and intervention programs.^14^

**Figure 1.**
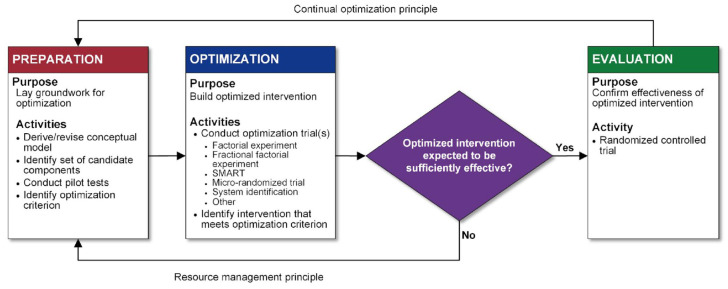
Flow chart of the multiphase optimization strategy (MOST) framework as described by Collins^15^ Reproduced with permission from Springer Nature.

MOST is used to optimize interventions in several steps (Figure 1). In the preparation phase, “candidate” intervention components are identified and developed based on theory and a review of existing evidence. Formative evaluation and piloting of the components are also completed. During the optimization phase, short-term, randomized experiments are conducted, typically using factorial designs, in which every combination and level of each candidate component is independently randomly assigned. The factorial design allows researchers to rigorously examine main and interaction effects, and increase efficiency and statistical power (see the works of Collins^15^ and Dziak et al.^16^ for more information on factorial designs). During the evaluation phase, the optimized intervention is evaluated in an RCT, consisting only of those combined components that were shown to be effective or important in the optimization phase. Most evaluations of childhood obesity preventive interventions resemble this evaluation phase, without having completed the optimization phase. Only after the optimized intervention is found to be effective in the context of an RCT is it ready for release/dissemination as a new intervention to the public. If the optimized intervention is not effective, the process would begin again in the preparation phase.

This article describes the design of the Healthy Bodies Project, an optimization trial of a childhood obesity preventive intervention constructed using MOST. We describe the candidate components and the study design that were used as part of the optimization phase, along with study outcomes and measures. The goal was to select components with detectable effects on our key outcomes, in order to build a highly efficacious (i.e. results in significant behavioral changes) and highly efficient (i.e. includes only successful components) behavioral intervention to prevent childhood obesity.

## Methods

### Preparation phase: conceptual framework and development of the intervention components

The Healthy Bodies Project was designed as a multicomponent, childcare-, and family-based preventive intervention with the overarching goal of reducing preschool children’s obesity risk by improving their healthy eating knowledge, physical activity patterns, and behavioral self-regulation skills. The first goal of the preparation phase was to use evidence from the literature to inform the conceptual model and select candidate intervention components. The second goal was to pilot test the feasibility of the components and the study design. Development of the study was guided by social cognitive theory.^17^

**Figure 2.**
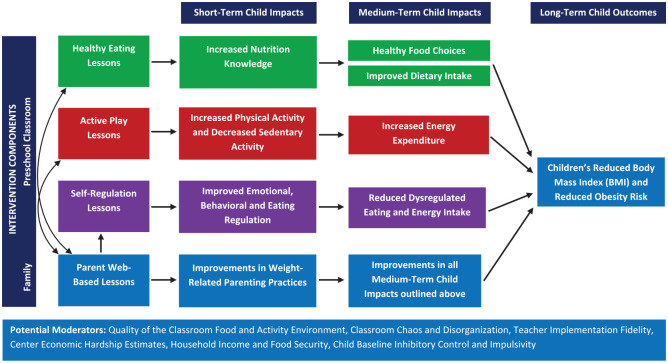
The Healthy Bodies Project conceptual model.

The conceptual framework appears in Figure 2, which outlines the behavior change techniques and potential mechanisms of change. We hypothesized that three separate intervention components—healthy eating, active play, and self-regulation classroom curricula—would each result in short-term impacts on weight-related targets, including children’s healthy eating knowledge, physical activity, and self-regulation of emotions, behaviors, and eating. Furthermore, we hypothesized that an additional component, parents’ engagement with web-based lessons, would bolster the effects of the classroom-based intervention on child outcomes. We did not expect to see significant changes in children’s BMI over the short course of the active portions of the intervention, which lasted 11 weeks. However, we hypothesized that changes in children’s healthy eating knowledge, physical activity, and self-regulation would potentially have long-term effects on reducing future obesity risk, through effects on the medium-term mechanisms of change outlined in Figure 2, which were not directly measured.

### Developing the candidate intervention components

**Figure 3.**
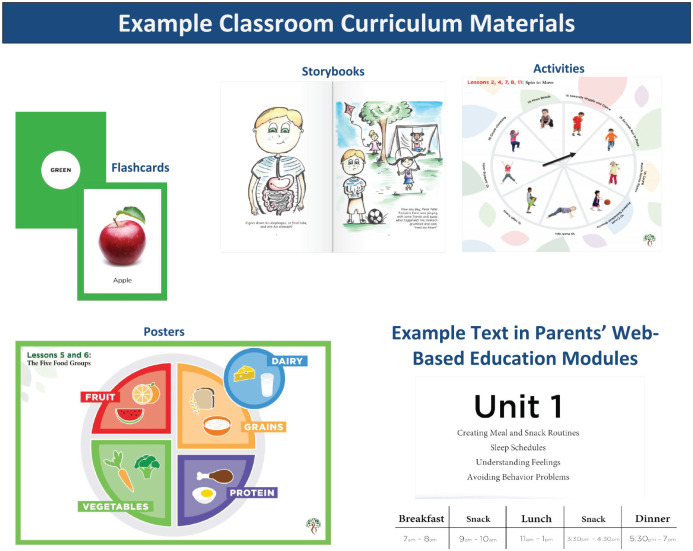
An example of materials used in classroom curricula and images used in the web-based parent education component.

Classroom curricula were designed to be implemented by preschool teachers. Lessons were designed to build on one another, with basic concepts discussed first, and subsequent lessons progressing to more demanding and skill-building challenges. Table 1 provides an overview of each classroom curriculum and their lessons, and example materials appear in Figure 3.

**Table 1.** List of lesson topics for each classroom curriculum tested in the optimization trial.

Healthy eating curriculum	
Lesson	Lesson topics/activities
Lesson 1: Creating a healthy restaurant	• Work together to create a healthy restaurant • Explore the various items and job responsibilities involved in creating a healthy restaurant
Lesson 2: GO foods	• Learn to identify healthy foods (GO foods) • Understand that healthy foods are good for our bodies
Lesson 3: WHOA foods	• Learn to identify foods that are not very healthy (WHOA foods) • Understand that less healthy foods should be not be eaten very often
Lesson 4: Mabel the mouse	• Review GO and WHOA foods • Categorize pictures of GO and WHOA foods • Choosing GO foods
Lesson 5: Food groups	• Learn the names of the five food groups • Learn to categorize foods into the five food groups
Lesson 6: Healthy plate	• Learn about building a healthy plate that includes all of the food groups
Lesson 7: Building a healthy menu	• Explore healthy eating with pretend play/Creating a healthy menu for the classroom restaurant
Lesson 8: Rainbow on my plate	• Explore the different colors of fruits and vegetables
Lesson 9: Try for five!	• Learn how many fruits and vegetables to eat each day
Lesson 10: Marvin the mouse	• Review of GO and WHOA foods • Categorize pictures of foods as GO foods and WHOA foods • Practice choosing GO foods
Lesson 11: Open your healthy restaurant!	• Open a healthy restaurant in the classroom using information learned over the previous 10 lessons
Active play curriculum	
All lessons	• Children will participate in 30 min of moderate-vigorous active play • Stretching exercises • Deep breathing and cool down after physical activity
Week 1	• Overview of the Move Your Body Game and Movement Cube • Practicing stretching • Practicing deep breathing
Week 2	• Movement spinner with various active movements • Animal poses
Weeks 3 and 7	• Movement cube and movement sticks • Olympics
Weeks 4 and 10	• Movement spinner • Animals in motion
Weeks 5, 8, and 11	• Movement cube • Sports day
Weeks 6 and 9	• Movement cube • Drop and move
Self-regulation curriculum	
Lesson 1: Calm down time	• Behavioral regulation—identifying feelings and emotions • Learn what it feels like to be excited and full of energy • Learn what it feels like to be calm and relaxed • Calm down strategies
Lesson 2: How I feel today	• Behavioral regulation—identifying feelings and emotions • Learn to communicate feelings to others • Learning about the classroom calm down spot
Lesson 3: Learning to wait	• Behavioral regulation—practicing waiting strategies
Lesson 4: Turtle race	• Behavioral regulation—inhibitory control • Behavioral regulation—practicing waiting strategies
Lesson 5: Follow the leader	• Behavioral regulation—inhibitory control and controlling impulses • Behavioral regulation—practicing waiting strategies
Lesson 6: Relaxing animal stretches (yoga poses)	• Relaxing animal stretches as a calm down strategy • Behavioral regulation—inhibitory control and controlling impulses • Behavioral regulation—waiting strategies
Lesson 7: Parts of my body that help me eat	• Eating regulation • Learning the parts of the body involved in digestion • Understanding what it feels like to be hungry and full
Lesson 8: How do our bodies feel when we are hungry/full?	• Eating regulation—understanding how hunger and fullness feels
Lesson 9: Why do you eat? Why do you stop eating?	• Eating regulation—reasons why we may start and stop eating
Lesson 10: What happens if you eat too much?	• Eating regulation—what happens if you eat too much
Lesson 11: Feeling just right	• Eating regulation—learning to eat until your body feels just right

Children in all experimental conditions received a food literacy curriculum, which included 27 lessons (including an introductory lesson) that introduced children to a new fruit or vegetable from A to Z each. Each lesson contained information on how and where the food grows, and why it is good for our bodies. Parents across all conditions received access to web-based parent resources (e.g. tip sheets) related to the food literacy curriculum. The food literacy curriculum was designed as an inactive, engagement component to provide some education to all children. We did not expect this curriculum to impact any of the short-term study outcomes, including healthy eating outcomes. None of the food literacy curricular content was focused on the specific outcomes that were targeted in the healthy eating curriculum, such as the food groups needed to build a healthy meal.

The healthy eating classroom component included 11 lessons that focused on improving children’s food knowledge ( e.g. identifying energy- versus nutrient-dense foods), and their ability to make healthy food choices for meals and snacks. The curriculum had a restaurant theme, and was designed to progressively build skills each week that would allow children to open a “healthy restaurant” by the last lesson.

The active play classroom component consisted of 11 lessons that included active games designed to increase children’s moderate-to-vigorous physical activity by ∼30 min during the school day. Each lesson consisted of a warm-up activity, an active game, a stretching activity, and a cool down breathing exercise. The curriculum included visual aids to promote high-energy movement and stretching. Teachers also had access to online how-to videos that demonstrated each activity. Language used throughout the lessons emphasized play.

The self-regulation classroom component consisted of 11 lessons focused on improving children’s self-regulation in three developmental domains: emotional regulation (*e.g.* identifying and talking about feelings), behavioral regulation (*e.g.* improving inhibitory control and waiting patiently), and eating-related regulation (*e.g.* recognizing hunger and satiety cues). In emotion regulation lessons, children learned to identify how they and others may feel in various situations. In behavioral regulation lessons, children practiced inhibitory control by learning a set of distraction strategies known to be an important aspect of behavioral regulation.^18,19^ Eating regulation lessons were adapted from an eating regulation curriculum described by Reigh et al.,^20^ designed to: (a) improve preschool children’s knowledge of digestion, hunger and fullness, (b) improve children’s short-term energy regulation, and (c) reduce eating in the absence of hunger.

Parent web-based lessons were created to provide parents with guidance on supporting (at home) the skills and knowledge that children gained from the classroom curricula (see Table 2 for an overview). Each lesson was approximately 2–3 min in length and packaged together in five education modules containing three or four lessons each. All lessons contained high-resolution images and animations that reflect the racial, ethnic, and occupational diversity of families in the United States (see Figure 3, e.g., of content).

**Table 2.** Parent education lesson topics, content, and targeted child outcomes.

Lesson	Lesson topics	Lesson content	Target child outcome
Module 1	1. Establishing meal and snack routines 2. Sleep schedules 3. Fostering emotion regulation/understanding feelings 4. Fostering behavior regulation/avoiding behavior problems	• Importance of establishing routines at home • Establishing meal, snack and sleep routines and consistency for health and behavior • The importance of helping children to identify and name feelings and emotions • Supporting children’s emotion regulation • Importance of giving children choices to help reduce behavior problems and avoid tantrums	Improve children’s emotional and behavioral regulation
Module 2	1. Healthy eating basics (fruits and veggies) 2. Grains, protein, and dairy 3. Healthy beverages 4. Healthy snacking	• Increasing acceptance and intake of a variety of fruits and vegetables • Importance of including whole grains, lean proteins, and low-fat dairy for a healthy diet • Importance of limiting sugar-sweetened beverages • Offering healthy snacks as part of a regular mealtime routine • Importance of making snacks “mini-meals” as opposed to treats	Improve children’s acceptance and intake of a variety of healthy foods
Module 3	1. Healthy shopping on a budget 2. Healthy choice at the store 3. Parent modeling of healthy eating 4. Picky eating	• Making healthy choices at the store while keeping a budget in mind • Ways to save money while shopping each of the five food groups • Making healthy shopping easy and affordable • Importance of parent modeling to build healthy eating habits for the family • Ways to help children learn to try and like new foods	Improve availability of healthy foods in the home while sticking to a budget, and increase children’s acceptance of those foods
Module 4	1. Mealtime environment 2. Stress-free mealtimes 3. Division of responsibility in feeding/avoiding mealtime struggles 4. Portion control	• Tips for making mealtime more pleasant for the entire family • Eating together as a family • Dealing with stressful situations to make mealtimes more enjoyable • Using mealtimes to build healthy family relationships • Child feeding strategies to help children realize and pay attention to hunger and fullness cues	Improve children’s behavioral and eating regulation
Module 5	1. Child physical activity 2. Parent physical activity	• Importance of physical activity for children • Ways to incorporate physical activity into daily routines • Importance of physical activity for parents	Increase children’s physical activity by at least 30 min each day

### Design of the optimization trial

The optimization trial incorporated a 2^4^ between-pre-existing units, complete factorial design in which children were nested in classrooms, and classrooms were nested in childcare centers. As shown in Table 3, classrooms were randomized to one of 16 distinct experimental conditions that varied based on whether the following candidate intervention components were received or not received: (a) healthy eating classroom component, (b) active play classroom component, (c) self-regulation classroom component, and (d) enhanced parent education. The food literacy classroom component was the fifth, core component that all classrooms received; the efficacy of this component was not tested in the optimization trial. Given the homogeneity of the childcare centers that we aimed to recruit, we did not attempt to balance randomization based on any specific cluster-level covariates. Restricted allocation was used to maintain balance (equal numbers of classrooms per experimental condition) by ignoring conditions during randomization once they reached the maximum number of clusters (six to seven classrooms/condition).

**Table 3.** Experimental conditions in the 2^4^ factorial design.

Condition	Intervention components				
	Core food literacy curriculum	Healthy eating curriculum	Active play curriculum	Self-regulation curriculum	Enhanced parent education
1	ON	ON	ON	ON	ON
2	ON	ON	ON	ON	OFF
3	ON	ON	ON	OFF	ON
4	ON	ON	ON	OFF	OFF
5	ON	ON	OFF	ON	ON
6	ON	ON	OFF	ON	OFF
7	ON	ON	OFF	OFF	ON
8	ON	ON	OFF	OFF	OFF
9	ON	OFF	ON	ON	ON
10	ON	OFF	ON	ON	OFF
11	ON	OFF	ON	OFF	ON
12	ON	OFF	ON	OFF	OFF
13	ON	OFF	OFF	ON	ON
14	ON	OFF	OFF	ON	OFF
15	ON	OFF	OFF	OFF	ON
16	ON	OFF	OFF	OFF	OFF

Note: All classrooms receive the core food literacy curriculum, which was not expected to impact any primary outcomes. This component was not tested in the optimization trial.

### Recruitment

Recruitment was targeted at center-based childcare programs in rural and semi-rural counties in Pennsylvania. Preferred characteristics included being open for full-day care, providing meals and snacks in the classroom, and serving at least 50% of Head-Start eligible families. To maximize the potential for change in child outcomes, we also targeted centers with a lower-quality care rating (3 or less on the 4-point Keystone Stars rating of childcare performance standards), given the positive association between childcare quality and children’s social and cognitive outcomes.^21,22^ Once centers were deemed eligible, in-person meetings with directors were scheduled to describe the study. Teachers were then contacted for an in-person meeting in which the study was described and signed consent was obtained. Randomization occurred, by research project managers, after teachers consented to the study. When there were multiple classrooms within a center, all classrooms were assigned to the same configuration of healthy eating, active play, and self-regulation curricula components, and only differed in terms of receiving or not receiving the parent web-based lessons. This decision was made to reduce contamination, facilitate coordination of activities within centers, and accommodate transitions between classrooms among some children.

Children were required to be aged 2–5 years at the start of the trial. Children were excluded from the study if they were younger than 3 years by January of the academic year, did not attend preschool during days/times that intervention lessons were taught, or if they had medical or developmental conditions that affected their ability to complete the research assessments. Caregivers were excluded if they were not responsible for feeding their preschool child at least 50% of the time or if they were not fluent in English. To avoid nesting within families, data from one randomly selected child per family was included. Because randomization occurred at the classroom level, all children participated in all classroom activities, but data were only collected from children with consent to participate. The study was approved by the Penn State Office for Research Protections and Institutional Review Board (CATS #2116).

### Study timeline and procedures

The optimization phase of the study was conducted between October 2017 and March 2020. The study was designed to occur over a 10-month period, which included teacher training, baseline assessments, classroom observations, and implementation of the 27-week food literacy curriculum. The four candidate components were implemented over 11 weeks beginning in January of each year. Child assessments were conducted at baseline (∼November), mid-intervention (∼January/February), post-intervention (∼March/April), and at 3-month follow-up (∼June). Physical and sedentary activity were the only measures collected at mid-intervention, on days that the active play curriculum was implemented. Teachers and parents completed web-based or paper-and-pencil surveys at baseline and post-intervention. Research staff and Penn State University Extension educators conducted two coaching sessions for the core food literacy curriculum, and two sessions per experimental curriculum assigned (0–6 visits), which were used to increase implementation fidelity. As such, these research staff were not blinded to experimental condition. During coaching visits, the classroom environment was observed, including teachers’ interactions with children and use of classroom materials.

### Measures

Data collection with children occurred in childcare centers during normal hours. Study team members, blinded to classroom condition, conducted individual research assessments with each child.

#### Primary child outcomes

Children’s nutrition knowledge and food choices were measured using two instruments. The *Food Knowledge Procedure* is an adaptation of the Placemat Protocol.^23^ Children were shown six picture sheets and were asked to first choose their favorite foods and beverages, and then to make a healthy pretend lunch by picking two items that are good for their body from each set of pictures. In the *Snack Selection Testing Protocol*,^24^ children were shown picture cards with pairs of energy-dense and nutrient-dense foods. Children were first asked to choose a food for snack, and then to choose the food that “helps you grow big, strong and healthy.” Responses were summed to create a score to indicate the number of energy-dense and nutrient-dense foods children identified or selected.

Children’s time (minutes) spent in physical and sedentary activity was measured using the *Actigraph wGT3X*, during a normal school day, on three separate weekdays. Monitors were worn on belts (attached by research staff) and placed at the hip. Teachers removed monitors at the end of the day, and delivered them to the research office using a courier service. Activity level was adjusted for wear time.

Child behavioral regulation was assessed using several measures via teacher and parent reports, direct testing of children, and observer ratings. Teachers and parents completed subscales from the Children’s Behavior Questionnaire (short version)^25^ that measured: (1) anger/frustration, (2) impulsivity, and (3) inhibitory control. Children completed three trials of the *Walk A Line Slowly* task,^26^ in which the child was asked to walk down a “path,” comprised a 2 frac12 inch × 6-foot strip of tape on the floor, as slowly as they can, and even slower than the previous trial. The length of time (seconds) for each trial was recorded, with slower walk times indicating greater inhibitory control. In the *Pencil Tapping* task,^27^ the child was instructed to tap a pencil one time when the experimenter taps two times, and to tap two times when the experimenter taps one time. The number of incorrect/incorrect taps was recorded across 16 trials. *Choosing Dinky Toys* is a procedure from the preschool version of the Laboratory Temperament Assessment Battery^28^ in which children were told that they can choose two toys from a container filled with a variety of toys, but, once they choose a toy, they cannot return it for a new one. The amount of time it takes to choose a toy (seconds), the approximate number of toys touched, and rule violations were recorded. Finally, following each child assessment, experimenters rated children’s attention/impulse control during the assessment using the *Preschool Self-Regulation Assessment*.^29^ Response options ranged from 0 to 3; scores were averaged across items, with higher scores indicating higher levels of attention/impulse control. Children’s eating regulation was estimated using the *Eating in the Absence of Hunger Questionnaire for Children and Adolescents: Parent Report of Child*,^30^ an assessment of parents’ perceptions of the degree to which children eat in response to negative affect (e.g. “feeling sad or depressed”), fatigue/boredom (e.g. “feeling tired”), and external stimuli (e.g. “because the food looks, tastes, or smells so good”). Response options range from 1 (never) to 5 (always), with mean scores indicating higher reports of children’s eating in the absence of hunger.

#### Secondary child outcomes

Children’s age- and sex-specific BMI (kg/m^2^) percentiles and *z*-scores^31^ were calculated using weight and height measured by trained research assistants. Children’s appetitive behaviors were measured using the Children’s Eating Behavior Questionnaire,^32^ a parent-report measure of eight dimensions of children’s eating behaviors, including satiety responsiveness (i.e. referring to the ability stop eating based on perceived fullness), slowness of eating, and food responsiveness. Response options range from 1 (never) to 5 (always).

#### Additional child, parent and family measures and potential covariates

In addition to family sociodemographic information (e.g. income, education, and family size), parents provided information on a variety of measures, including their own height and weight; food parenting practices; children’s picky eating behaviors;^33,34^ availability of fruits, vegetables, whole grains, and dairy foods in the home; children’s requests for each of those foods; parent and family physical activity levels^35^ and media use;^36,37^ child sleep patterns;^38^ and household food security.^39^ These measures were used to explore differential responses to various components of the project.

#### Potential moderators: childcare environment and implementation fidelity

Observations of the classroom environment included the food environment (*e.g.* posters depicting healthy foods), mealtime environments (*e.g.* teachers’ use of coercive feeding practices), and the physical activity environment (*e.g.* space and equipment that support physical activity). A modified version of the *Confusion, Hubbub and Order Scale*^40^ was used to measure classroom chaos. Measures of implementation fidelity include participation rates for teacher training, and the degree to which teachers implemented classroom materials as intended.

## Results

### Sample size determination

Power estimates were computed using approaches described by Dziak et al.^16^ The significance level (α) was set to .05 and power was set at 80%. We assumed a correlation of 0.65 between children’s pretest and posttest measures, and a mean cluster (classroom) size of 15 children and standard deviation of 2. Because we planned to recruit childcare centers from a fairly homogeneous pool in terms of family income, we estimated that the variability between centers on child outcomes (intraclass correlation coefficient), would be < 0.10, using the childcare centers’ state-level quality rating as a covariate to account for center-level variability. To detect a given main effect of size *d* = 0.20 under these assumptions, the optimization trial needed a sample size of about 1380 children in 92 classrooms (estimating 15 children/classroom, and 5–6 classrooms/condition). Given an estimate of attrition between 5% and 10%, and to ensure adequate power, we chose to recruit ∼100 classrooms (from a pool of more than 500 classrooms in our target counties).

When the study concluded in March 2020, a total of 1397 preschool children, nested within 113 classrooms (of 119 randomized; 95% retention) and 63 childcare centers, were enrolled; this resulted in ∼12 children per classroom and ∼7 classrooms per experimental condition. Of the 1947 parents that were recruited to participate in the parent portion of the study, 1172 consented to participate (60% participation). A total of 799 parents provided survey data (68% response rate), and 373 parents (of 541 randomized; 69% response rate) completed web-based education modules.

### Analytical plan

To examine the efficacy of each candidate component, intent-to-treat analyses can be utilized, using data from all eligible children and parents who participated in the study, regardless of absences or attrition. The main effects of each component can be examined using a full effects hierarchical linear model (random slopes with restricted maximum-likelihood estimation), with assessment periods nested within children, children nested in classrooms and classrooms nested within centers. Effect coding should be used to dichotomize each component, representing the presence (1) or absence (–1) of each intervention component (see Kugler et al.^41^). All main effects and possible interaction effects should be evaluated to examine which intervention components are efficacious, either on their own or in combination with other intervention components. The main effects of an individual component can be evaluated by comparing the mean of a target outcome for children across all of the experimental conditions in which a component is turned on, and the mean for children across all conditions in which the component is turned off. Using the Healthy Bodies Project as an example, to determine the main effect of the healthy eating classroom component, we would examine the difference in the mean changes in food knowledge between children across Conditions 1–8 combined (healthy eating = ON) and children across Conditions 9–16 combined (healthy eating = OFF; see Table 3). Interactions can be explored by evaluating mean differences in food knowledge outcomes when additional components are turned on or off.

To further enhance the precision of statistical estimates, the hierarchical linear model can include children’s race, age, and gender as Level 1 covariates. To minimize the extent to which variation in treatment effects are attributable to classroom (cluster level) characteristics, Level 2 covariates can also include teachers’ implementation fidelity, classroom environment (food, mealtime, and physical activity), and classroom chaos. To account for center-level aggregate characteristics, models can include the center quality rating and an estimate of economic hardship faced by families (percentage of Head Start-eligible families) as a pretest covariate. To account for the repeated measures design, subject-level pretest, posttest, and follow-up scores on target outcomes for each component can be included as separate timepoints (repeated measures) in the model, or models examining mean differences in posttest and follow-up scores across conditions can be adjusted for pretest scores; both approaches are described in the work of Dziak et al.^16^ The authors note that inclusion of a center-level pretest covariate comes at the sacrifice of scarce degrees of freedom. Because we were not interested in change between the posttest and follow-up periods in this optimization trial, the focus should be on modeling linear change between the pre- and posttests, and between the pretest and follow-up. Because the study was not powered to detect moderation effects, moderation analyses ( e.g. teachers’ implementation fidelity) should be considered exploratory.

## Discussion

### Selection and refinement of intervention components

Findings from the optimization trial will be used to refine and select intervention components. Following approaches described by Kugler et al.,^42^ components would first be considered important and would be included in the candidate intervention if they yielded an effect size around *d* = 0.20. Given that MOST is an inherently exploratory technique, subsequent decisions about which component to include are made based on additional factors, such as the *p*-value of intervention effects < *d* = 0.20 that were determined to be conceptually/theoretically important in the preparation phase, the intervention component’s unique or redundant impact on improvements in a specific target outcome, or whether the intervention component was deemed important for a subgroup of children. Details on the decision-making process in MOST can be found in the work of Collins.^15^

Although child BMI change was not a primary study outcome, it would be prudent to estimate the combined effects of each component on children’s pre–post change in BMI and conditional weight gain, specifically for children with overweight or obesity at baseline. Findings from a meta-analysis of interventions with a parent component designed to reduce obesity in early childhood showed that short-term effects on children’s BMI outcomes were small (*d* = 0.10).^43^ We note that if all 4 of our intervention components are shown to have a similar effect on child BMI, we would expect the combined effect size to be at least *d* = 0.10 and potentially as high as *d* = 0.40.

To date, few obesity prevention or intervention studies have been designed using MOST. At the time of submission of this manuscript, a search of the literature using the terms “multiphase optimization strategy” and “obesity” yielded 12 published papers describing the design of or results from obesity prevention or intervention studies using the MOST framework. Of these 12 published papers, three described studies targeting preschoolers or parents of preschoolers,^44–46^ one described a study targeting parents of elementary-age students,^47^ and one described a study targeting adolescents.^48^ Albeit a crude, non-exhaustive search, these findings suggest that the large majority of multicomponent childhood obesity prevention programs may have been designed and implemented with an all-in, kitchen sink approach. Adopting a MOST approach to the design of childhood obesity prevention programs should result in RCTs that are efficient, and packaged with only the most efficacious components. The MOST framework will allow us to select the most promising configuration of intervention components to be tested in an RCT of the Healthy Bodies Project.

## Project oversight

An investigators committee provided oversight for the design and implementation of this trial. The committee was comprised of study investigators listed on this article (LF, RN, RB, KLK, KCK, and JS), research staff, Extension educators, an Extension coordinator, and early childhood education experts. Study investigators listed on this article will provide oversight for data analysis and interpretation, and dissemination of the trial findings. The funder will not be involved in any audits of the trial.
